# Beneficial effects of PGC-1α in the substantia nigra of a mouse model of MPTP-induced dopaminergic neurotoxicity

**DOI:** 10.18632/aging.102357

**Published:** 2019-10-21

**Authors:** Yingqing Wang, Chun Chen, Wanling Huang, Maoxin Huang, Juhua Wang, Xiaochun Chen, Qinyong Ye

**Affiliations:** 1 Department of Neurology, Fujian Institute of Geriatrics, The Affiliated Union Hospital of Fujian Medical University, Fuzhou, Fujian, China; 2 Clinical Medicine, Xiangya School of Medicine, Central South University, Changsha, Hunan, China; 3 Key Laboratory of Brain Aging and Neurodegenerative Diseases, Fujian Key Laboratory of Molecular Neurology, Fujian Medical University, Fuzhou, China

**Keywords:** Parkinson’s disease, C57BL mice, mitochondria, MPTP, PGC-1α

## Abstract

Background: Mitochondrial dysfunction and oxidative stress are closely associated with the pathogenesis of Parkinson’s disease. Peroxisome proliferator-activated receptor γ coactivator 1 alpha (PGC-1α) is thought to play multiple roles in the regulation of mitochondrial biogenesis and cellular energy metabolism. We recently reported that altering PGC-1α gene expression modulates mitochondrial functions in N-methyl-4-phenylpyridinium ion (MPP^+^) treated human SH-SY5Y neuroblastoma cells, possibly via the regulation of Estrogen-related receptor α (ERRα), nuclear respiratory factor 1 (NRF-1), nuclear respiratory factor 2 (NRF-2) and peroxisome proliferator-activated receptor γ (PPARγ) expression. In the present study, we aimed to further investigate the potential beneficial effects of PGC-1α in the substantia nigra of 1-methyl-4-phenyl-1,2,3,6-tetrahydropyridine (MPTP) treated C57BL mice.

Methods: The overexpression or knockdown of the PGC-1α gene in the mouse model of dopaminergic neurotoxicity was performed using a stereotactic injection of lentivirus in MPTP-treated male C57BL/6 mice. Mice were randomly assigned to one of 6 groups (n=24 per group): normal saline (NS) intraperitoneal injection (i.p.) (con); MPTP i.p. (M); solvent of the lentivirus striatal injection (lentivirus control) + MPTP i.p. (LVcon+M); lentivirus striatal injection + MPTP i.p. (LV+M); LV-PGC-1α striatum injection + MPTP i.p. (LVPGC+M); and LV-PGC-1α-siRNA striatal injection + MPTP i.p. (LVsiRNA+M). Intraperitoneal injections of MPTP/NS were conducted two weeks after lentivirus injection.

Results: We found significant improvement in motor behavior and increases in tyrosine hydroxylase expression in the substantia nigra (SN) in the brains of mice in the LVPGC+M group. The opposite tendency was observed in those in the LVsiRNA+M group. The expression of superoxide dismutase (SOD) in the SN region was also consistent with the changes in PGC-1α expression. Electron microscopy showed an increasing trend in the mitochondrial density in the LVPGC+M group and a decreasing trend in the M and LVsiRNA+M groups compared to that in the controls.

Conclusions: Our results indicated that PGC-1α rescues the effects of MPTP-induced mitochondrial dysfunction in C57BL mice.

## INTRODUCTION

As the second most common adult-onset neurodegenerative disease next to Alzheimer’s disease, idiopathic Parkinson’s disease (PD) is characterized by α-synuclein accumulation and the progressive loss of dopaminergic neurons in the human substantia nigra pars compacta (SNpc). The inhibition of the electron transport chain (ETC) and mitochondrial dysfunction have been implicated in the pathogenesis of PD. The first line of evidence was obtained from the description of ETC complex I (CI) deficiency in a postmortem study of PD [1, 2]. Neurotoxins, including 1-methyl-4-phenyl-1,2,3,6-tetrahydropyridine (MPTP) and rotenone, were found to inhibit CI activity [3]. Peroxisome proliferator-activated receptor γ (PPARγ) coactivator 1 alpha (PGC-1α) is a key regulator of mitochondrial biogenesis and cellular energy metabolism in response to extracellular changes [4, 5]. Recent studies have highlighted important roles of PGC-1α in neurodegenerative diseases. A genome-wide meta-analysis identified a general downregulation of PGC-1α-responsive gene transcription in the SN of PD patients [6]. Increased neuron vulnerability to neurotoxicity [7] and loss of dopaminergic neurons [8] were found in PGC-1α knockdown mice. The overexpression of PGC-1α in mouse neurons also resulted in increased resistance against MPTP-induced nigrostriatal degeneration and ETC activity [9], suggesting the potential significance of PGC-1α in the treatment of PD.

Our previous *ex vivo* study using adenovirus transfection to manipulate PGC-1α gene expression in N-methyl-4-phenylpyridinium ion (MPP^+^) -treated neuroblastoma cells showed changes in several mitochondrial functions. These changes occurred in the mitochondrial membrane potential, ATP production, cytochrome C release, and H_2_O_2_ production. The effects of PGC-1α, manifesting as the regulation of downstream transcriptional factors, were also identified. Estrogen-related receptor α (ERRα) and nuclear respiratory factor 1 (NRF-1) were found to be the key co-factors in cellular protection [10, 11]. To shed further light on the therapeutic benefits of PGC-1α, we investigated the effects of manipulating its expression in MPTP-treated C57BL mice in the present study. For bidirectional comparison, the lentivirus system was used both for PGC-1α overexpression and knockdown, and the engineered lentivirus preparations were injected into the striatum to establish two types of mouse models. Using these models, we primarily focused on behavioral changes, dopaminergic neuron survival, and mitochondrial changes in the SN after MPTP treatment.

### Declarations

We used male C57BL/6 mice. All animal experiments were carried out under the guidelines of the Animal Care and Use Committee of Fujian Medical University. The animal experimentation protocols were approved by the ethics review committee of Fujian Medical University.

## RESULTS

### LV-PGC-1α and LV-PGC-1α-siRNA were widely distributed in the SN region

**Figure 1:**
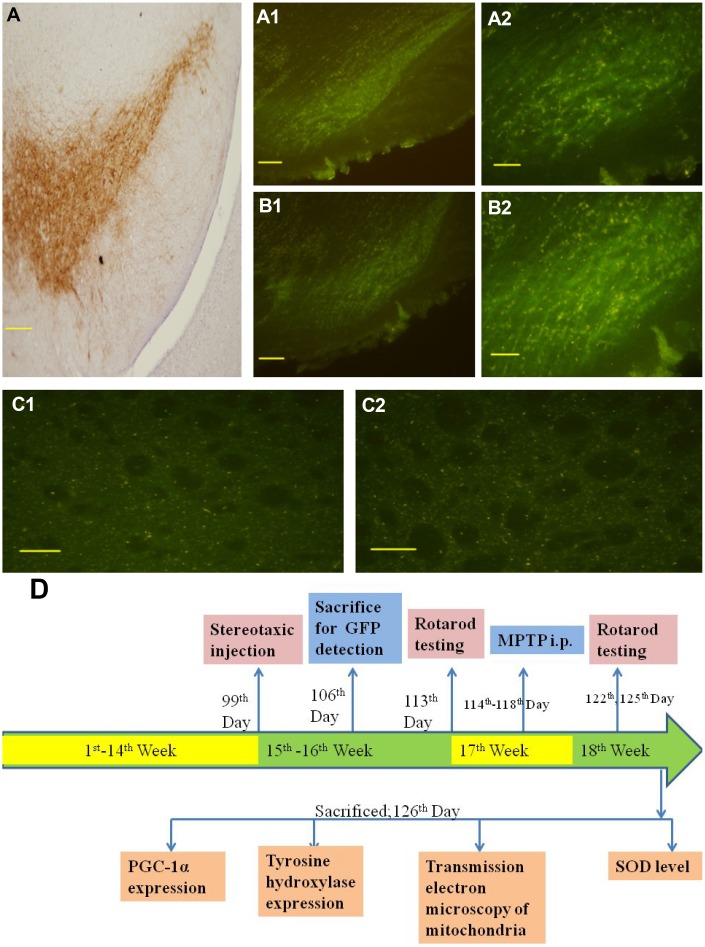
**Distribution of lentivirus in the SN, striatum region and the representative schematic.** (**A**) shows immunohistochemical staining of the substantia nigra in the control group. (**A1** and **A2**) show successful LV-PGC-1α infection in the substantia nigra of mice. (**B1** and **B2**) show successful LV-PGC-1α-siRNA infection of the substantia nigra of mice. (**C1** and **C2**) show the striatum infected with LV-PGC-1α and LV-PGC-1α-siRNA, respectively. (**D**) shows a schematic representation of the experimental paradigm. Scale bar: 20 μm. Magnification: ×200 for **A1**, **B1**; ×400 for **A**, **A2**, **B2**, **C1**, **C2**.

To observe whether lentivirus was transmitted from the striatum to the substantia nigra, one week after infection, GFP was detected to prove successful lentivirus transfection. GFP fluorescence was observed in cells throughout the substantia nigra and striatum (Figure 1), which demonstrated that lentivirus could be transported from the striatum to the substantia nigra and successfully transfected into cells.

### Changes in PGC-1α expression in the SN

To determine whether PGC-1a was overexpressed or knocked down in the substantia nigra, we detected the protein and mRNA levels of PGC-1a in the substantia nigra.

**Figure 2:**
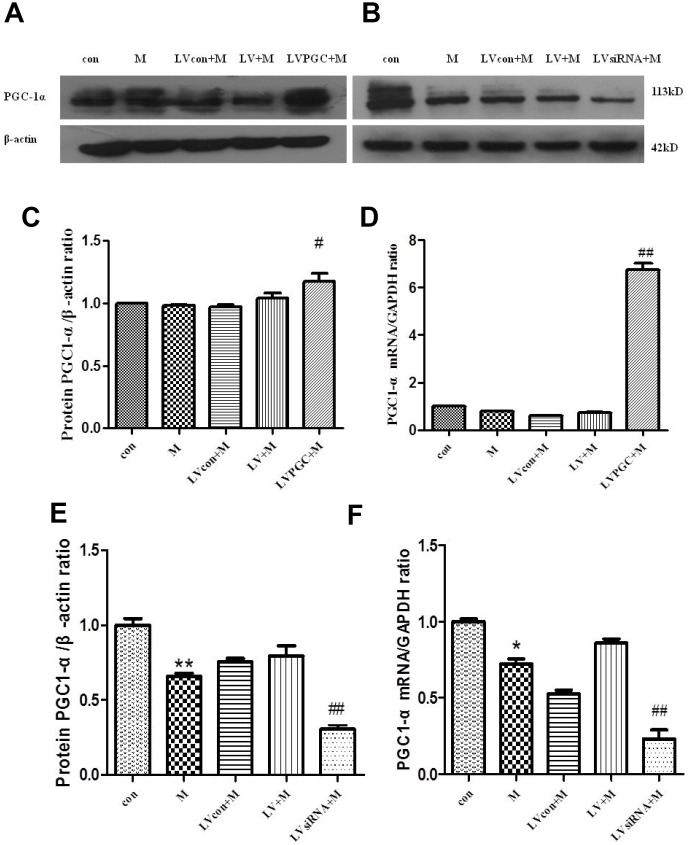
**Expression of PGC-1α in the SN of C57BL mice.** (**A**, **B**) PGC-1α protein expression patterns; (**C**, **D**) PGC-1α protein expression patterns after overexpression, detected using western blotting and real-time PCR; (**E**, **F**) PGC-1 α protein expression after knockdown, detected using western blotting and real-time PCR; Quantification of the results of three experiments are presented as the means ± SD. The groups were con (con group), M (MPTP group), LVcon+M (solvent of lentivirus+MPTP group), LV+M (lentivirus+MPTP group), LVPGC+M (LV-PGC-1α+MPTP group), LVsiRNA+M (LV-PGC-1αsiRNA+MPTP group); #P<0.05, ##P<0.01 compared with the LV+M group, **P<0.01, *P<0.05 compared with the con group.

In the overexpression analysis, western blotting (Figure 2A, 2C) showed that PGC-1α expression in the LVPGC+M group was 1.13 times higher than that in the LV+M group (P <0.05), while no differences were found between the M and con groups. Real-time PCR (Figure 2D) showed that PGC-1α mRNA levels in the LVPGC+M group were 8.64 times higher than those in the LV+M group (P <0.01), and those of the M group showed no significant differences compared with those of the con group, which showed a consistently positive trend of protein expression. In the knockdown analysis, western blotting (Figure 2B, 2E) showed a significant reduction in PGC-1α expression levels when treated with MPTP and LV-PGC-1α-siRNA (M vs. con, 26.67%, P<0.01; LVsiRNA+M vs. LV+M, 62.72%, P<0.01). The same trend was found in the mRNA levels of PGC-1α (Figure 2F) (M vs. con, 27.84%, P<0.05; LVsiRNA+M vs. LV+M, 73.12%, P<0.01). Thus, PGC-1α was successfully overexpressed and knocked down in the mouse SN at both the protein and mRNA levels using our overexpression and siRNA PGC-1α constructs. Additionally, MPTP could alter PGC1-α expression levels, but not as much as the LV-PGC-1α and LV-PGC-1α-siRNA.

### PGC-1α improves MPTP-induced behavioral abnormalities

A successful mouse model of MPTP-induced dopaminergic neurotoxicity is the basis for the next experiment. The success of the model can be judged by the difference in behavior before and after modeling. The rotarod test is a widely used behavioral test used to assess balance and body coordination in mice [12, 13]. This test rapidly provides easily interpretable results for investigators. The equipment is simple and easy to operate.

**Figure 3:**
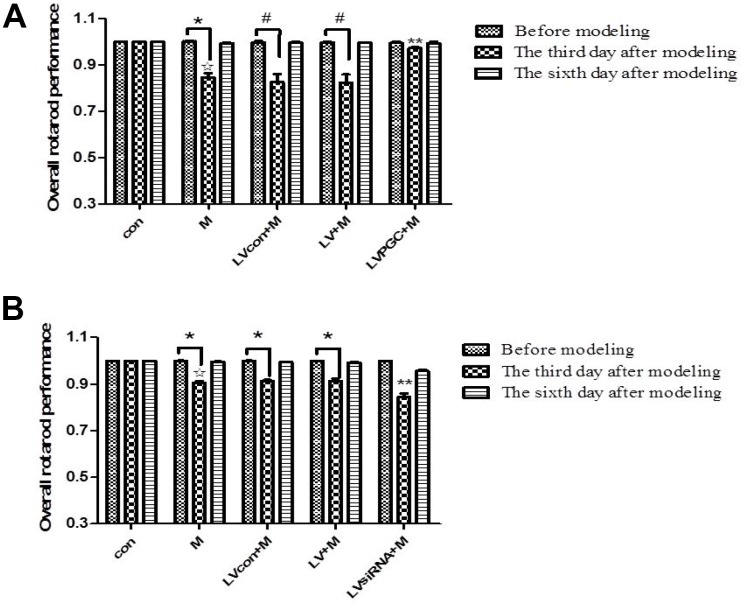
**Mouse behavioral changes before and after MPTP administration**. (**A**, **B**) The overall performance of mice in the rotarod test one day before injection and three days and six days after injection. The results of the three experiments are quantified as the means ± SD. The groups were con (con group), M (MPTP group), LVcon+M (solvent of lentivirus+MPTP group), LV+M (lentivirus+MPTP group), LVPGC+M (LV-PGC-1α+MPTP group), and LVsiRNA+M (LV-PGC-1αsiRNA+MPTP group);*P<0.01, #P<0.05 compared with before injection; ☆P<0.01 compared with the con group; **P<0.01 compared with the LV+M group.

The time the mouse stayed on the rod was recorded and plotted against the correlated rotarod speed. The area under the curve was calculated to represent the overall performance of individual mice (rotarod test scores). In the overexpression experiments, the results (Figure 3A) showed that three days after MPTP administration, the rotarod test scores of the M group, the LVcon+M group, and the LV+M group decreased by 15.30% (P <0.01), 17.14% (P < 0.05), and 17.38% (P <0.05), respectively, compared with the scores on the day before MPTP administration. Comparing groups on the third day after MPTP administration showed that the performance of the M group decreased by 15.28% (P<0.01) compared with that of the con group, and the performance of the LVPGC+M group increased by 18% (P<0.01) compared with that of the LV+M group. In the knockdown experiment, the results (Figure 3B) showed that three days after MPTP administration, the rotarod test scores of the M group, the LVcon+M group, and the LV+M group decreased by 9.50% (P <0.01), 8.93% (P < 0.01), 8.90% (P <0.01), respectively, compared with the scores on the day before MPTP administration. Comparing the groups on the third day after MPTP administration showed that the performance of the M group decreased by 9.43% (P<0.01) compared with that of the con group, and the performance of the LVsiRNA+M group decreased by 7.50% (P<0.01) compared with that of the LV+M group.

### PGC-1α attenuates MPTP-induced tyrosine hydroxylase loss in the mouse substantia nigra

**Figure 4:**
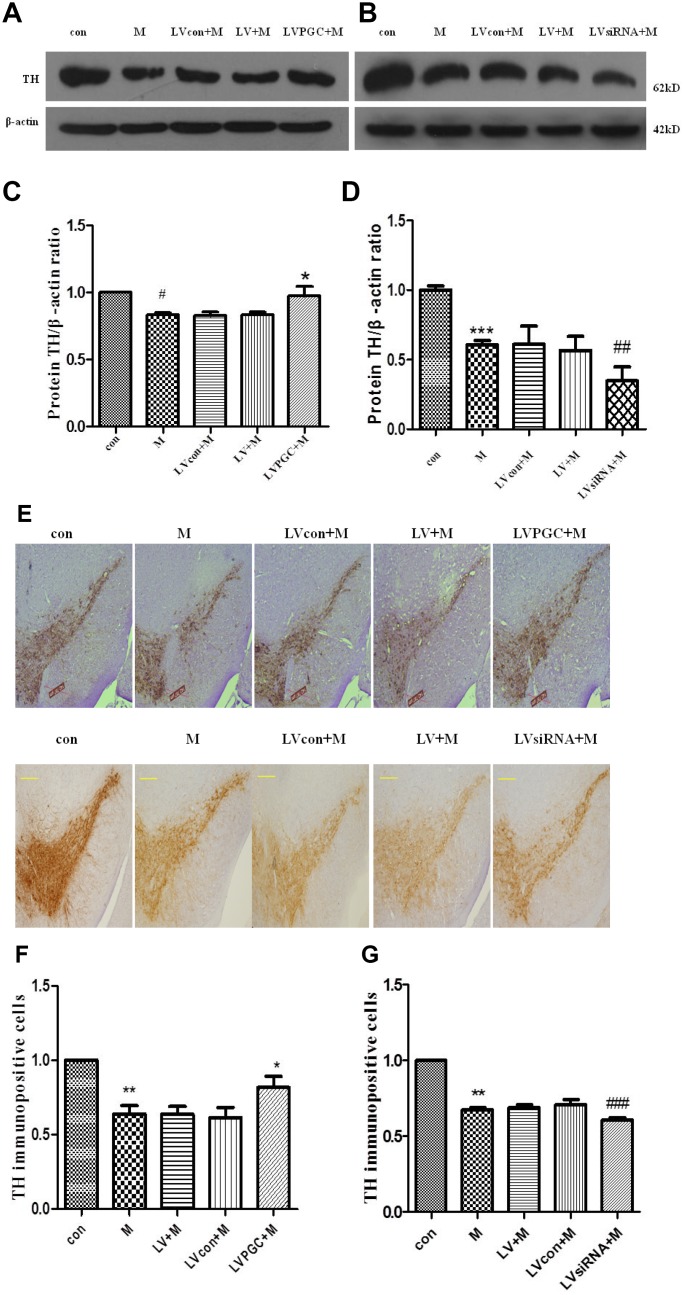
**Expression of TH and TH immunopositive cells in the substantia nigra of C57BL mice.** (**A**–**D**) TH protein expression detected using western blotting; (**E**–**G**) TH immunopositive cells (scale bar, 20 μm. magnification, ×400). Quantification of the results from the three experiments are represented as the means ± SD. The groups were con (con group), M (MPTP group), LVcon+M (solvent of lentivirus+MPTP group), LV+M (lentivirus+MPTP group), LVPGC+M (LV-PGC-1α+MPTP group), LVsiRNA+M (LV-PGC-1αsiRNA+MPTP group);*P<0.05, ##P<0.01, ###P<0.001, compared with the LV+M group; #P<0.05, ***P<0.001, **P<0.01 compared with the con group.

The success of the model can also be judged by the decrease in the dopaminergic neurons in the substantia nigra. Dopamine is an inhibitory and excitabory neurotransmitter in the central nervous system that is mainly synthesized by dopaminergic neurons in the substantia nigra of the midbrain and transported to the striatum via the substantia nigra–striatum pathway. TH, the key rate-limiting enzyme in the dopamine synthesis process, expression levels are consistent with the dopamine content in the nigrostriatal. Detecting the expression of TH can reflect the number and functional status of dopamine neurons, so this marker can be used to detect whether the MPTP-induced dopaminergic neurotoxicity model is successful [14]. The results of western blotting in the overexpression studies (Figure 4A, 4C) revealed that the TH levels in the SN were 16.70% lower in the M group than those in the con group (P <0.05), while those in the LVPGC+M group were 16.6% higher than those in the LV+M group (P <0.05). In the knockdown studies (Figure 4B, 4D), there was a significant decrease in TH expression in the M group compared to that in the con group (M vs. con, 39.31%, P<0.001). TH expression in the LVsiRNA+M group decreased by 38.61% compared with that in the LV+M group (P<0.01).

Immunohistochemical staining (Figure 4E) demonstrated positive brown TH staining in the SN. Dopamine-containing neurons were identified by their TH content. In the overexpression experiments (Figure 4F), the number of TH immunopositive cells in the SN of mice in the M group decreased by 36.13% compared to that in the con group (P<0.01), and the number in the LVPGC+M group increased by 32% compared to that in the LV+M group (P<0.05). In the knockdown studies (Figure 4G), TH expression levels in the M group decreased by 32.5% compared with those in the con group (P<0.01), and levels in the LVsiRNA+M group decreased by 13.95% compared with that in the LVsiRNA+M group (P<0.001). By immunostaining, we found that TH-positive neurons showed a similar tendency to that of the TH protein in the SN. A prominent decrease in TH neuron loss induced by MPTP was observed. Taken together, the expression levels of PGC1-α determine the TH protein level and the number of TH immunopositive cells.

### Analysis of mitochondrial morphological changes after PGC-1α intervention

PGC-1α is one of the major regulators of mitochondrial biological origin and energy metabolism in cells. PGC-1α can compensate for neuronal mitochondrial damage by increasing the number of mitochondria and protecting the morphological structure of mitochondria.

**Figure 5:**
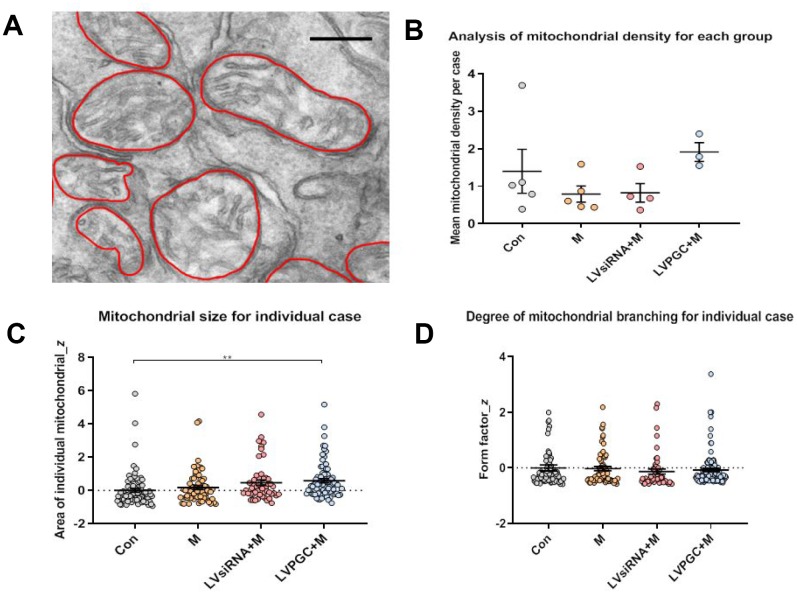
**Electron microscopy observation of the destruction of mitochondrial structure in SN neurons.** (**A**) Using electron microscopy (EM), SN images were collected from the group of con (number of mice, n=5), M (number of mice, n=5), LVPGC+M (number of mice, n=3), and LVsiRNA+M (number of mice, n=3). A total of 281 mitochondria were manually outlined and analyzed using QuPath software. Scale bar, 300 nm. (**B**) The average level of mitochondrial density was calculated for individual mice, which showed a higher trend in mitochondrial density in the LVPGC+M group and a lower trend in the M and LVsiRNA+M group compared to that in controls (P>0.05). (**C**) Mitochondrial size in the LVPGC+M group was significantly increased compared with that in control mice (P>0.01). (**D**) The degree of mitochondrial branching was also compared via the calculation of individual mitochondrial form factors.

To examine whether PGC-1α could protect mitochondria from MPTP intoxication, we investigated the morphological changes in mitochondria one week after MPTP administration using transmission electron microscopy (EM). (Figure 5A). There was an increased trend in the mitochondrial density in the LVPGC+M group and a decreasing trend in the M and LVsiRNA+M groups compared to that in the controls (Figure 5B). However, these trends did not reach statistical significance, possibly due to the small sample size and individual diversity within experimental animals. We found a significantly larger mitochondria area in the SN of LVPGC+M mice than in the control mice, which could possibly be due to enhanced mitochondrial biogenesis by PGC-1α (Figure 5C). There was no difference in the degree of mitochondrial branching between groups (Figure 5D).

### SOD level in the substantia nigra

The neuroprotective effects of PGC-1**α** may be related to the inhibition of ROS production, the increase in antioxidant enzymes and the protection of mitochondrial function. Superoxide dismutase (SOD) is the main antioxidant enzyme in mitochondria, and it can transform oxygen free radicals such as OH, H_2_, O2 and other oxygen radicals into nontoxic hydrogen peroxide.

**Figure 6:**
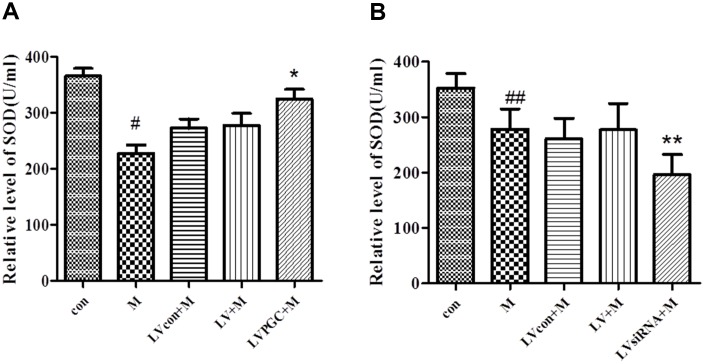
**Effects of PGC-1α on the level of superoxide dismutase in a mouse model of Parkinson’s disease (PD) induced by MPTP.** (**A**, **B**) Activity of SOD in the SN region of C57BL/6 mice with PGC-1α overexpression or knockdown. Quantification of the results of the three experiments are presented as the means ± SD. The groups were con (con group), M (MPTP group), LVcon+M (solvent of lentivirus+MPTP group), LV+M (lentivirus+MPTP group), LVPGC+M (LV-PGC-1α+MPTP group), and LVsiRNA+M (LV-PGC-1αsiRNA+MPTP group);*P<0.01, **P<0.05 compared with the LV+M group; #P<0.01, ##P<0.05 compared with the con group.

The WST-1 method was used to measure SOD concentration in SN homogenates from each group. In the overexpression studies (Figure 6A, Table 1), the levels of SOD induced by MPTP were 27.60% lower than those in the con group (P<0.01), while those in the LVPGC+M group were 17% higher than those in the LV+M group (P<0.01). In the knockdown studies (Figure 6B, Table 1), the SOD level showed a 21.15% decrease in the SN region after MPTP treatment compared with that in the con group (P<0.05). The SOD level in the LVsiRNA+M group was 29.26% lower than that in the LV+M group (P<0.05). The data above showed that MPTP treatment reduced total SOD activities, and the expression levels of PGC-1α determined the SOD activities.

**Table 1:** SOD level shown as mean value ± standard deviation.

**Groups**	**con**	**M**	**LVcon+M**	**LV+M**	**LVPGC+M/ LVsiRNA+M**
**SOD level (U/ml)**					
**Over-expression**	365.58±13.59	227.92±14.52	277.04±21.66	272.47±16.29	324.65±17.21
**Knock-down**	352.90±25.90	278.24±36.43	277.30±47.28	260.88±36.91	196.17±36.51

## DISCUSSION

The primary aim of the present study was to attain a better understanding of the role of PGC-1α in the SN of an MPTP-treated dopaminergic neurotoxicity model, with a particular focus on beneficial effects against SN neuronal loss and mitochondrial dysfunction.

To establish an animal model with both dopaminergic neurotoxicity and mitochondrial dysfunction, a subacute MPTP treatment was implemented in C57BL/6 mice. The classic neurotoxin MPTP penetrates the blood-brain barrier and is then biotransformed by monoamine oxidase (MAO) to MPP^+^ in the brain. MPP^+^ specifically suppresses the activity of mitochondrial ETC complex I, leading to further ETC dysfunction, increased oxidative stress, and neuronal death. Behavioral performance and SN neuronal loss were investigated before and after the intervention to validate the model. Mice showed impaired rotarod performance three days after MPTP administration, including poor coordination and reduced endurance. However, this decrease was not observed on the sixth day after MPTP administration. It is interesting that the reduction in TH levels in the SN continued until the seventh day after MPTP administration, indicating that the recovery in rotarod performance was not due to the recovery of dopaminergic neurons. This result may be due to the existence of compensatory mechanisms, which first mask the existence of Parkinson’s disease before the appearance of the first clinical symptoms and then delay the onset and aggravation of motor behavioral abnormalities. A number of factors are involved in these compensatory processes: the intrinsic properties of dopaminergic nigrostriatal neurons, neuronal plasticity, nonclassic DA volume transmission in the striatum, and different inputs to the SNc [15, 16]. Spontaneous recovery in behavioral performance has been reported in MPTP-treated mice [17]. In our study, performance on the third day may be due to acute cell injury after modeling, which leads to functional damage. After that, a compensatory mechanism is initiated that improves behavioral performance on the sixth day.

Human peroxisome proliferator-activated receptor γ (PPARγ) coactivator 1α (PGC-1α), together with PGC-1β and the PGC-related coactivator PRC, constitute the transcriptional coactivators of the peroxisome proliferator activated receptor (PGC) family. To meet energy demands under different physiological and developmental conditions, PGC-1α activity is activated via phosphorylation by a range of upstream signal factors. In recent rodent studies, the role of AMPK/ PPAR/PGC-1α signaling has been highlighted as having beneficial effects through the promotion of mitochondrial biogenesis, leading to neural protection [18, 19]. The overexpression of PGC-1α rescued mitochondrial defects through an increase in mitochondrial biogenesis and the maintenance of mitochondrial morphology in neurons [20, 21]. Furthermore, PGC-1α null mice showed increased vulnerability toward the neurodegenerative effects of MPTP. Consistent with the abovementioned results, in our study, knocking down PGC-1α in the SN of MPTP-treated mice aggravated the toxic effects of MPTP, including a further loss of TH-positive neurons and impaired motor performance. It is notable that TH, the key rate-limiting enzyme involved in dopamine synthesis, was higher in the remaining neurons, indicating the potential role of PGC-1α in the maintenance of neuronal function to balance dopamine content. This result could also explain the improved motor symptoms in the PGC-1α-overexpressing mice.

The activation of PGC-1α initiates the transcription cascade via the coactivation of several downstream regulators, including ERRα and mitofusin 1 and 2 (MFN1/MFN2), whose levels are altered due to mitochondrial fission and fusion. Increased PGC-1α was reported to directly regulate mitochondrial dynamics, mainly by regulating the expression of fission and fusion genes in dopaminergic neurons of rat and human skeletal muscle [22, 23]. We observed enlarged mitochondria in PGC-1α-overexpressing mice, possibly due to increased mitochondrial biogenesis and mitochondrial fusion. However, comparing mitochondrial branching did not show evidence to support the increase in mitochondrial fusion. The limitation of using two-dimensional EM analysis added extra difficulties in the interpretation of the mitochondrial morphological changes. Three-dimensional image acquisition and a larger sample size will be employed in the further investigation of mitochondrial morphology changes.

PGC-1α is also associated with the generation of ROS and the expression of antioxidant enzymes [9, 24]. Mitochondrial DNA mutation, energy depletion, calcium overload, mitophagy, and apoptosis could be related to the overload of ROS [25]. This vicious circle is responsible for neuronal degeneration in the human midbrain [26]. PGC-1α reduces ROS generation by regulating the expression and activity of ROS-detoxifying enzymes [27, 28]. SOD is one of the main antioxidant enzymes in mitochondria, converting oxygen radicals into nontoxic hydrogen peroxide. The overexpression of SOD reduced neurotoxicity in mouse SN neurons [29]. Conversely, mice deficient in CuZn-SOD and Mn-SOD genes were more susceptible to MPTP [30, 31]. PGC-1α plays an important role in regulating the expression and activity of the mitochondrial antioxidant enzyme SOD2 in myocardial cells, endothelial cells and neurons [28, 32, 33]. Previously, we found that the overexpression of PGC-1α reduced the release of cytochrome C and inhibited H_2_O_2_ production in neuroblastoma [10]. In this study, the SOD level was lower after MPTP treatment in the SN of mice and was significantly increased in the SN of PGC-1α-overexpressing mice. In contrast, an even lower level of SOD expression was observed in the SN after PGC-1α-knockdown. It remains unclear whether this positive response originates from neurons themselves or from surrounding glial cells because there has been evidence of the ability of astrocytes to provide healthy mitochondria to neurons in response to increased oxidative stress [34].

## CONCLUSIONS

In a series of *ex vivo* studies, we validated the protective effects of PGC-1α via manipulating its expression in human neuroblastoma cells with mitochondrial dysfunction [10, 11]. We then established similar mouse models to further assess the potential of PGC-1α effects *in vivo*. In MPTP-treated mice, PGC-1α inhibited MPTP-induced oxidative damage. This effect included the upregulation of SOD levels, the maintenance of mitochondrial structure, and improvements in SN neuron degeneration**.** The specific regulatory pathway involving PGC-1α and other factors protecting C57BL/6 mice against MPTP warrants further study.

## MATERIALS AND METHODS

### Animals

Male C57BL/6 mice (weight 25–28 g, 14–16 weeks old, SLAC) were kept under a conventional 12-hour light–dark cycle in a temperature-controlled (20 ± 2°C) room with free access to food and water.

Mice were randomly assigned to the following groups (n=24 per group): normal saline (NS) intraperitoneal injection (i.p.) (con); MPTP i.p. (M); Solvent of lentivirus (lentivirus control) striatal injection + MPTP i.p. (LVcon+M); Lentivirus striatal injection + MPTP i.p. (LV+M); LV-PGC-1α striatal injection + MPTP i.p. (LVPGC+M); and LV-PGC-1α-siRNA striatal injection + MPTP i.p. (LVsiRNA+M). Intraperitoneal injections of MPTP/NS were conducted two weeks after lentivirus injection.

### Lentivirus production and stereotaxic injection

Lentivirus containing green fluorescent protein (GFP) and the *PGC-1α* overexpression/siRNA constructs was engineered by GeneChem Technology. Viral stocks were thawed on ice and diluted to 2×10^8^ TU/ml (LV-PGC-1α) and 5×10^8^ TU/ml (LV-PGC-1α-siRNA) with a lentivirus solvent. Mice were anesthetized with 10% chloral hydrate (400 mg/kg, i.p.) and positioned on a stereotactic head frame (Stoelting). Solvent lentivirus (4μl), LV-PGC-1α (4μl), and LV-PGC-1α-siRNA (2.5μl) were stereotactically injected into the striatum at a speed of 0.25 μl/min. Geometry-oriented coordinates of the mouse striatum were as follows: anterior, 0.5 mm; lateral, 2.2 mm; ventral, 3.4 mm; bregma as reference. After injection, the needle was left in place for an additional five minutes and slowly withdrawn (protocol adapted from [35]).

### MPTP treatment

Two weeks after striatal injections, mice were subjected to a total cumulative dose of 150 mg/kg MPTP i.p. (M0896, Sigma) for five consecutive days (30 mg/kg per day). The same amount of NS was injected into the con group to be used as the control. Whole brain samples were collected seven days after the last MPTP administration and were subdivided by different experimental procedures.

### Rotarod test

Rotarod testing was performed on an automated 5-lane rotarod unit (YLS-4C, Yiyan Science and Technology). All mice were pretrained for three consecutive days with two trials per day with 15-minute intervals between each trial. Formal tests were conducted before and on the third and sixth days after MPTP injections and were repeated three times per day with a minimum interval of 15 minutes between tests. The duration of each rotation was recorded, and the results were analyzed per the suggested protocol [12, 13, 36].

### Western blot analysis

Seven days after the last MPTP injection, mice were anesthetized as mentioned above and intracardially perfused with 30 ml of 0.9% cold NS. Each SN was dissected on prechilled glass plates and lysed with RIPA lysis buffer (1% sodium deoxycholate, 0.1% SDS, 1 mM sodium orthovanadate, 50 mM sodium fluoride, and 1 mM EDTA). The supernatants were collected and processed using the standard Bradford assay (Beyotime).

Forty-five micrograms of denatured protein was loaded per lane and resolved by 8% SDS-PAGE (sodium dodecyl sulfate-polyacrylamide gel electrophoresis) for 90 minutes at 80 V. The separated proteins were transferred onto PVDF (polyvinylidene fluoride) membranes (Millipore, Carrigtwohill, Ireland) for 2 hours at 200 mA with Bradford reagent (Bio-Rad, Hercules, CA, USA). The membranes were blocked with 5% skimmed milk in 1×PBS (phosphate buffered saline) containing 0.05% Tween 20 (PBST) for 4 hours at room temperature. The following primary antibodies were used: anti-PGC-1α 1:1000 (EMD Millipore Billerica, MA, USA); anti-TH 1:1000 (1:1500 for suppression part) (EMD Millipore Billerica, MA, USA), anti-Actin (1:2000 Beyotime Company of Biotechnology Shanghai, China), which were contained in PBST and incubated at 4°C overnight. The membranes were washed three times in PBST for 10 minutes. Subsequently, the membranes were incubated for 1.5 hours in PBST containing secondary antibody conjugated to horseradish peroxidase (anti-rabbit IgG 1:2000 and anti-mouse IgG 1:2000, Beyotime Institute of Biotechnology, Shanghai, China). The immunoreactive bands were visualized and quantified using the enhanced chemiluminescence (ECL) detection kit (Millipore, USA).

### Quantitative real-time PCR

Total RNA extraction from SN homogenates was performed using the TRIzol method (Invitrogen). RNA was reverse-transcribed into cDNA using the RevertAidTM First Strand cDNA Synthesis Kit (K1621, Fermentas), and the amplification of cDNA was performed using the Fast Start Universal SYBR Green Master Mix (ROX) (Roche). Quantitative real-time PCR was carried out on the ABI Prism 7500 HT Sequence Detection System (Applied Biosystems) using the 59-nuclease assay for the indicated genes and the housekeeping gene GAPDH. The level of mRNA was compared by quantifying the ΔΔCt value as described in [10, 11]**.** The primer sequences were as follows: LV-PGC-1α forward, 5′-AAGCACTTCGGTCATCCCTG TC-3′; reverse, 5′-CGCACTTTCATCTTCACT GTCA TC-3′; LV-PGC-1α-siRNA forward, 5′-AGCACTTCG GTCATCCCTGTC-3; reverse, 5′-GCACTTTCATCTT CACTGTCATC-3′ and GAPDH forward, 5′-ACGGCA AGTTCAACGGCACA G-3′; reverse, 5′-GAAGACGC CAGTAGACTCCACGAC-3′.

### Immunohistochemistry

For immunohistochemical staining, mice were perfused with 30 ml of 0.9% cold normal saline and 30 ml of 4% paraformaldehyde (PFA). The SN of each mouse was postfixed with 4% PFA for another 24 hours at 4°C and processed with 30% sucrose solution for 24–48 hours at 4°C. Tissues were then cryo-sectioned at 8 μm thickness using a cryo-microtome (Leica).

Sections were dried at 37°C for an hour and immersed in 3% hydrogen peroxide (diluted in 100% v/v methanol) for 30 minutes. The sections were blocked with 3% normal goat serum for 30 minutes and incubated with rabbit anti-tyrosine hydroxylase (TH) antibody (1:400, Millipore) in a humidified chamber at 4°C overnight. This procedure was followed by incubation with biotinylated secondary antibody (anti-rabbit immunoglobulin G, 1:100) for one hour at room temperature. The sections were then labeled with streptavidin-peroxidase, 3,3-diaminobenzidine tetrahydrochloride dehydrate (DAB), and hematoxylin in sequence. After serial ethanol gradients (70%, 90% and 100% v/v) and xylene, the slides were mounted with Permount TM for storage. To measure the density of TH-positive cells in the substantia nigra, we performed counting as described by Bian M et al. [37]. One out of five sections and five sections were collected (n = 3 per group).

### Electron microscopy

The SN of each mouse was quickly dissected and processed with 3% glutaraldehyde, 1.5% paraformaldehyde, and 1.0 M PBS at 4°C per 24 hours each. This procedure was followed by postfixations in 1% osmium tetroxide and 1.5% potassium ferrocyanide at 4°C for 1.5 hours. After PBS washes, the samples were dehydrated in a graded series of ethanol (75%, 95%, 100% v/v) and embedded in an Epon-Araldite solution at 60°C for 72 hours. Sections were then cut at 100 mm thickness using an ultramicrotome (Leica) and imaged under an EM208 transmission electron microscope (Philips). For the analysis of EM images, individual mitochondria were manually outlined, and mitochondrial area and dimeter were measured using QuPath software (v.0.1.2). The total mitochondrial area per snap was divided by snap area to represent a comparative mitochondrial density value. The mitochondrial form factor was calculated to show the degree of mitochondrial branching [38] based on the following equation: *Form factor =* *Perimeter^2^/(4× π × area).* All data were transformed into *z* scores and normalized based on controls.

### ELISA measurement of SOD activity

Each mouse SN was dissected on prechilled glass plates, weighed, and soaked in 0.9% cold NS to prepare 10% tissue homogenates. The supernatants were collected after a ten minute centrifugation (3000 rpm) at 4°C and stored at −80°C. SOD activity was determined using the Superoxide Dismutase (SOD) WST-1 assay kit (Nanjing Jiancheng Bioengineering).

### Statistical analysis

All quantitative data were collected from at least three independent experiments. Data were analyzed using SPSS 17.0 statistical software. Real-time PCR data (Ct values) were converted into the 2^-△△Ct^ format for statistical analysis. Differences between the mean values were analyzed using one-way analysis of variance (ANOVA), and *P* < 0.05 was considered significant.
